# Transposition-Based Method for the Rapid Generation of Gene-Targeting Vectors to Produce Cre/Flp-Modifiable Conditional Knock-Out Mice

**DOI:** 10.1371/journal.pone.0004341

**Published:** 2009-02-05

**Authors:** Hilkka Turakainen, Jonna Saarimäki-Vire, Natalia Sinjushina, Juha Partanen, Harri Savilahti

**Affiliations:** 1 Program in Cellular Biotechnology, Institute of Biotechnology, Viikki Biocenter, University of Helsinki, Helsinki, Finland; 2 Program in Developmental Biology, Institute of Biotechnology, Viikki Biocenter, University of Helsinki, Helsinki, Finland; 3 Division of Genetics and Physiology, Department of Biology, University of Turku, Turku, Finland; University of Cincinnati, United States of America

## Abstract

Conditional gene targeting strategies are progressively used to study gene function tissue-specifically and/or at a defined time period. Instrumental to all of these strategies is the generation of targeting vectors, and any methodology that would streamline the procedure would be highly beneficial. We describe a comprehensive transposition-based strategy to produce gene-targeting vectors for the generation of mouse conditional alleles. The system employs a universal cloning vector and two custom-designed mini-Mu transposons. It produces targeting constructions directly from BAC clones, and the alleles generated are modifiable by Cre and Flp recombinases. We demonstrate the applicability of the methodology by modifying two mouse genes, *Chd22* and *Drapc1*. This straightforward strategy should be readily suitable for high-throughput targeting vector production.

## Introduction

In mouse, conditional gene knockout (cko) strategies have provided means to study the effects of gene inactivation in a tissue-specific manner and/or at a defined time period [1]. The most widely applied strategies for conditional mutagenesis take advantage of site-specific recombinases; particularly the Cre/loxP system of bacteriophage P1 [2] has been used extensively for the purpose [3].

Engineering the targeting vector construction is arguably one of the most time-consuming steps in cko strategies. Conventional methods for the construction of cko vectors include finding appropriate restriction enzyme cleavage sites in the genome, and several cloning steps to insert loxP sites as well as positive and negative selection markers into the construction. To complement the conventional methods, alternative strategies have recently been developed to construct cko targeting vectors. These strategies employ PCR in combination with homologous recombination (recombineering) [4]–[6], or they are based on the utilization of *in vitro* reactions of several DNA transposition systems, such as Ty1 [7], phage Mu [8]–[10], or Tn*5*/Tn*7* [11]. Although the currently available cko vector construction strategies are adequate for many transgenic projects, a general strategy that would further streamline the vector engineering procedure would be highly beneficial. In addition, the strategy should preferentially incorporate two commonly used site-specific recombination systems, Cre/loxP [2] and Flp/FRT [12], to allow versatile possibilities for the removal of selection cassettes in mouse ES cells or animals [3].

Mu DNA transposition reaction is one of the best-characterized transposition reactions [13], and a minimal version of it can be reproduced in *in vitro* conditions using MuA transposase, transposon DNA, and target DNA as the only macromolecular components [14]. Importantly, the relatively random transposon insertion spectrum allows near-saturating mutagenesis whereby insertions can be targeted to almost every residue in the target sequence [15]. The minimal *in vitro* reaction has recently been used in a variety of molecular biology, protein engineering, and genomics applications [16]–[24]. We have shown earlier that Mu *in vitro* transposition can be used to produce several types of mouse gene targeting constructions, including those generating null, hypomorphic, or conditional alleles [8]. This proof of principle study, employing mouse DNA subcloned in a plasmid vector, established the basic methodology and prompted us to examine possibilities for ever more advanced utilization of the technology.

Here, we describe a highly efficient Mu *in vitro* transposition-based approach to generate cko targeting vectors directly from BAC clones. By targeting the mouse *Cdh22* [25] and *Drapc1* [26] loci, we show that the strategy provides a straightforward means to produce conditional alleles.

## Results

**Figure 1:**
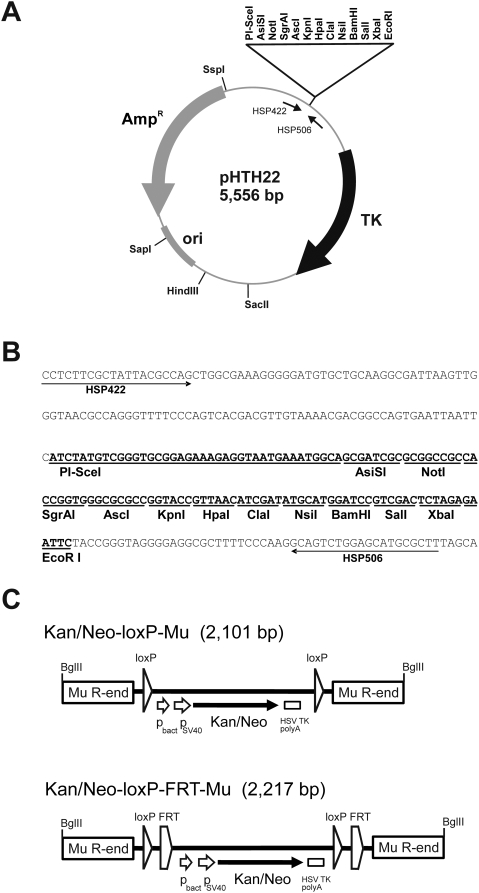
Details of the genetic tools used. (A) Structure and characteristic elements of the multicopy cloning vector pHTH22 with unique restriction sites indicated. The gene region encoding HSV thymidine kinase (TK) under the control of mouse phosphoglycerate kinase promoter and terminator is shown with a black curved arrow. The vector portion of the plasmid including the gene for ampicillin resistance (Amp^R^) and pUC19 origin of replication (ori) are shown with gray symbols (curved arrow and rectangle). (B) The nucleotide sequence of the polylinker and its flanking regions in pHTH22. The polylinker is shown in boldface with enzyme recognition sites underlined. The arrows indicate the binding sites of primers that were used for the confirmatory sequencing of genomic inserts' ends. (C) Structures of the Kan/Neo-loxP-Mu and Kan/Neo-loxP-FRT-Mu transposons. The transposons contain bacterial (p_bact_) and eukaryotic (p_SV40_) promoters (short arrows), a marker gene (Kan/Neo) conferring resistance to kanamycin in bacteria and G418 in eukaryotes (black arrow), and the HSV thymidine kinase polyadenylation (HSV TK polyA) signal (small rectangle). The loxP sites are indicated by triangles and FRT sites by pentagons. The rectangles in the transposon ends indicate 50 bp of Mu R-end DNA sequences in inverted orientation relative to each other. For the sake of clarity, the features are not in scale. The BglII sites in the ends are used to excise the transposons from their carrier plasmids. The sequences of the transposon-containing plasmids pHTH19 (Kan/Neo-loxP-Mu) and pHTH24 (Kan/Neo-loxP-FRT-Mu) are available upon request.

MuA transposase catalyzes transposon integration into any DNA in an *in vitro* reaction and produces essentially random distribution of transposon insertions along the target sequence [14]. We describe a strategy for the generation of cko targeting plasmids by employing two successive Mu transposition reactions to introduce a loxP site on one side of the exon of interest and a marker gene flanked by loxP sites and FRT sites on the other side of that exon. Initially, we constructed a universal cloning vector (pHTH22) suitable for essentially any cko project in mouse (Figure 1A). For negative selection in mouse ES cells [27], it contains the HSV TK gene flanked by the mouse PGK promoter and terminator. For cloning and linearization, it contains an array of unique restriction sites (Figure 1B), including several 8-cutter sites, and a homing endonuclease site (PI-SceI). We also constructed two selectable marker-containing mini-Mu transposons (Figure 1C). The first transposon, Kan/Neo-loxP-Mu, contains a bacterial promoter, the SV40 early promoter, the Kan/Neo resistance-encoding gene from Tn*5*, and the polyadenylation signal from HSV TK gene. This resistance cassette is flanked by two loxP direct repeats and embedded between two 50-bp inverted repeat Mu R-end sequences. The second transposon, Kan/Neo-loxP-FRT-Mu, contains two additional FRT sites as direct repeats. The functionality of the transposons, particularly Mu R-ends, was confirmed by *in vitro* transposition into an external target plasmid (data not shown). The functionality of the Cre/loxP and Flp/FRT recombination systems was tested by introducing appropriate transposon-containing plasmids into *E. coli* strains expressing Cre or Flp recombinase. As a result, all expected deletion derivatives of plasmids were formed, verifying the functionality of the two recombination systems (data not shown).

### Targeting vector for mouse *Cdh22* gene

**Figure 2:**
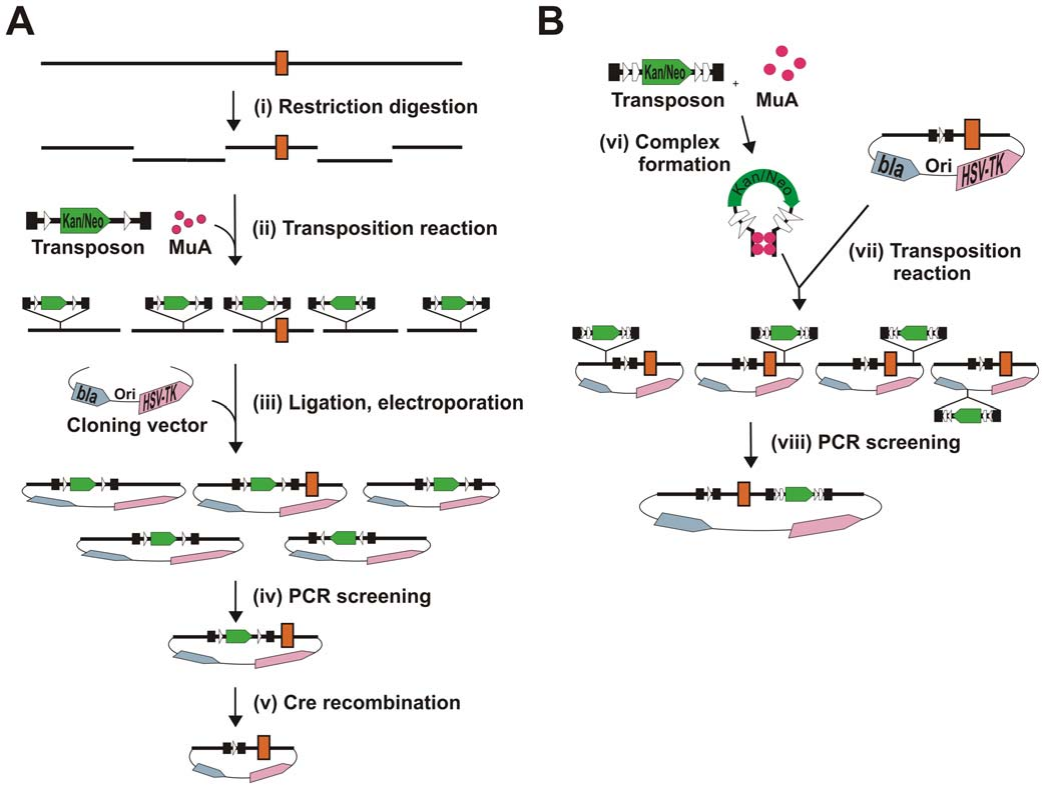
Flowchart for the construction of conditional knockout vectors. Part A. (i) an entire BAC clone is initially digested with an appropriate restriction endonuclease, and (ii) the ensuing fragment pool is then used as a target for the first transposition reaction. (iii) The fragment pool is subsequently ligated into a suitable vector plasmid, and those clones that include transposon-containing BAC fragments are selected using both the transposon marker and vector marker. Next, (iv) a plasmid clone that contains a transposon insertion in a desired location is identified by a PCR screen with appropriate primers, one transposon specific and the other target specific. (v) The chosen plasmid is then introduced into an *E. coli* strain that expresses Cre recombinase. The selectable marker is eliminated by Cre recombination *in vivo*, leaving a single loxP site in the construction. Part B. The second transposition reaction (vi) using pre-assembled transposition complexes (vii) introduces into the construction a marker gene that is flanked by loxP and FRT sites. (viii) As above, a suitable clone is screened by PCR to identify a plasmid, which contains the second transposon inserted in a suitable location and orientation on the opposite side (to the first transposon) of the exon of interest. Genomic DNA is shown with a black line, and the orange rectangle denotes an exon. The transposons are shown with black bars featuring the Kan/Neo cassette (green arrow), loxP sites (white triangles), FRT sites (white pentagons), and transposon ends (black rectangles).

**Figure 3:**
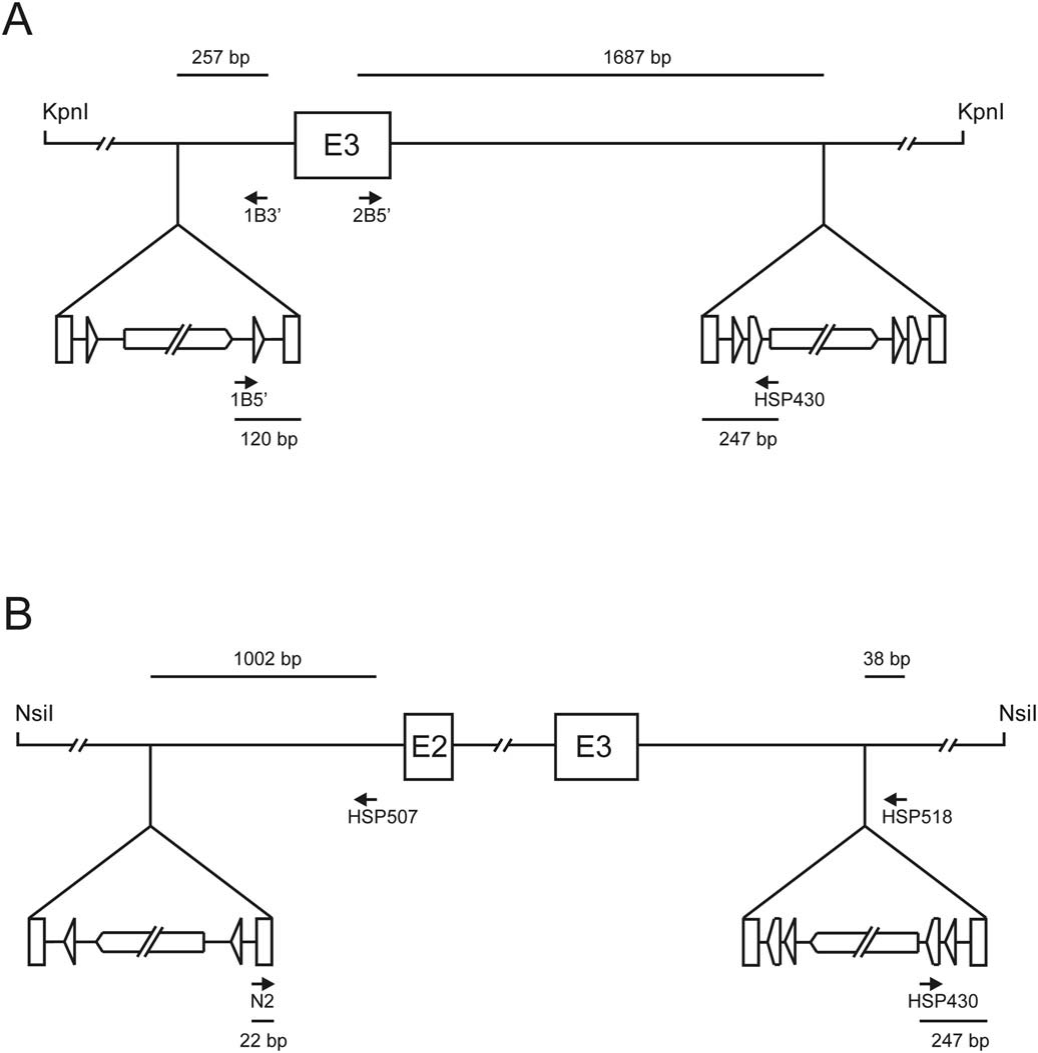
PCR screening of transposon-containing plasmid clones. (A) *Cdh22* locus. The first transposon insertion was screened using the primer pair 1B5′/1B3′. The second transposon insertion was screened using the primer pair 2B5′/HSP430. (B) *Drapc1* locus. The first transposon insertion was screened using the primer pair N2/HSP507. The second transposon insertion was screened using the primer pair HSP430/HSP518. In both panels, the relevant distances from the identified transposon insertion site to the 5′ ends of the primers are shown in base pairs (bp). For the sake of clarity, figure elements are not drawn in scale.

We applied the devised strategy (Figures 2) to generate a conditional *Cdh22* gene (MGI:1341843) knockout allele. As the initial transposition reaction target DNA, we used a KpnI digest of the identified BAC clone (110 kb), which contained the exon 3 of the mouse *Cdh22* gene in a 9 kb fragment. As the donor DNA, we used Kan/Neo-loxP-Mu transposon, containing the selectable Kan/Neo gene cassette between two loxP sites. Using selection for ampicillin and kanamycin resistance, transposition reaction products were cloned into the targeting vector as a pool, generating a plasmid library of ∼3,100 Amp^R^/Kan^R^ clones. We pooled 300 transformant clones in six pools, each containing 50 colonies, isolated plasmid DNA from these pooled samples, and screened the DNA by PCR for suitably located transposon insertions (Figure 3A). Three of these pools produced a prominent PCR product, the length of which fell within the desired size range (100–1,000 bp). Original clones from these three pools were then analyzed individually by colony PCR. Three clones, one from each pool, generated a PCR product that matched in size those products observed with pooled samples, indicating the presence of the transposon in the vicinity of the exon 3 in these three clones.

Most of the transposon DNA was then eliminated from these three integrant clones by introducing the plasmids into the *E. coli* strain expressing Cre recombinase. Site-specific recombination *in vivo* removed the selection cassette, leaving behind the Mu R-ends and one loxP site; and this was confirmed by sequencing. Sequencing also identified the exact location of the insertion in the selected three plasmid clones: 211, 368, and 462 bp upstream from the exon 3.

Next, the second transposon, Kan/Neo-loxP-FRT-Mu, with two additional FRT sites (“floxed” and “flrted” Kan/Neo gene cassette) was integrated *in vitro* into the construct that contained remnants of the first transposon 368 bp from the exon 3. In this case, we first assembled DNA transposition complexes by incubating the transposon with MuA transposase and then included the target DNA, followed by the addition of Mg^2+^ ions. The assembly of Mu transposition complexes is a slow process when compared to target capture and strand transfer steps of transposition. Thus, the use of pre-assembled complexes with a short incubation time thereafter prevents the target DNA from acting as a transposon, a formal possibility, as the target plasmid contains two Mu R-ends left over from the first integrated transposon. Transposition reaction products were then electroporated into *E. coli* DH10B cells, and proper clones were selected on plates containing ampicillin and kanamycin. We screened 80 transformants by colony PCR (Figure 3A), and six of the clones apparently contained a suitably located transposon, as indicated by a PCR product of appropriate size. One clone was selected, and subsequent sequence analysis confirmed a correct orientation and location of the second transposon 1,618 bp downstream from the exon 3.

### Targeting vector for mouse *Drapc1* gene

As another example, we generated a conditional knockout allele for Drapc1 gene (MGI:3513977). In the initial transposition reaction, we used an NsiI restriction digest of the BAC clone containing exons 2 and 3 as the target DNA, and Kan/Neo-loxP-Mu transposon as the donor. The desired NsiI fragment was 15.1 kb in length, containing the exons 2 and 3 with an intervening 2.8 kb intron and 5.2 kb upstream and 6.6 kb downstream regions. The reaction products were cloned into the targeting vector as a pool, generating a plasmid library of ∼6,700 Amp^R^/Kan^R^ transformants. The library was divided into forty pools (∼170 colonies per pool), and these pools were screened by PCR (Figure 3B). In this case, two of the pools generated a PCR product of appropriate size. Next, individual clones from one of the identified pools were analyzed by colony PCR for the respective transposon integration. A proper candidate clone was found; sequencing verified the transposon orientation and confirmed its location 1,220 bp upstream from the exon 2. Most of the transposon was again removed in the Cre-expressing *E. coli* strain. Next, the other transposon, Kan/Neo-loxP-FRT-Mu, was introduced by *in vitro* transposition as described for *Cdh22*. A total of 150 transformants were analyzed for PCR products as pools (Figure 3B), each pool containing ten transformants, and one of the pools generated a PCR product of appropriate size. The colonies from this pool were then analyzed individually by colony PCR, and a colony was identified that evidently was responsible for the abovementioned PCR product. Sequencing of this clone confirmed the correct orientation of the transposon and identified its exact location 964 bp downstream from exon 3.

### Functionality of the loxP and FRT sites

**Figure 4:**
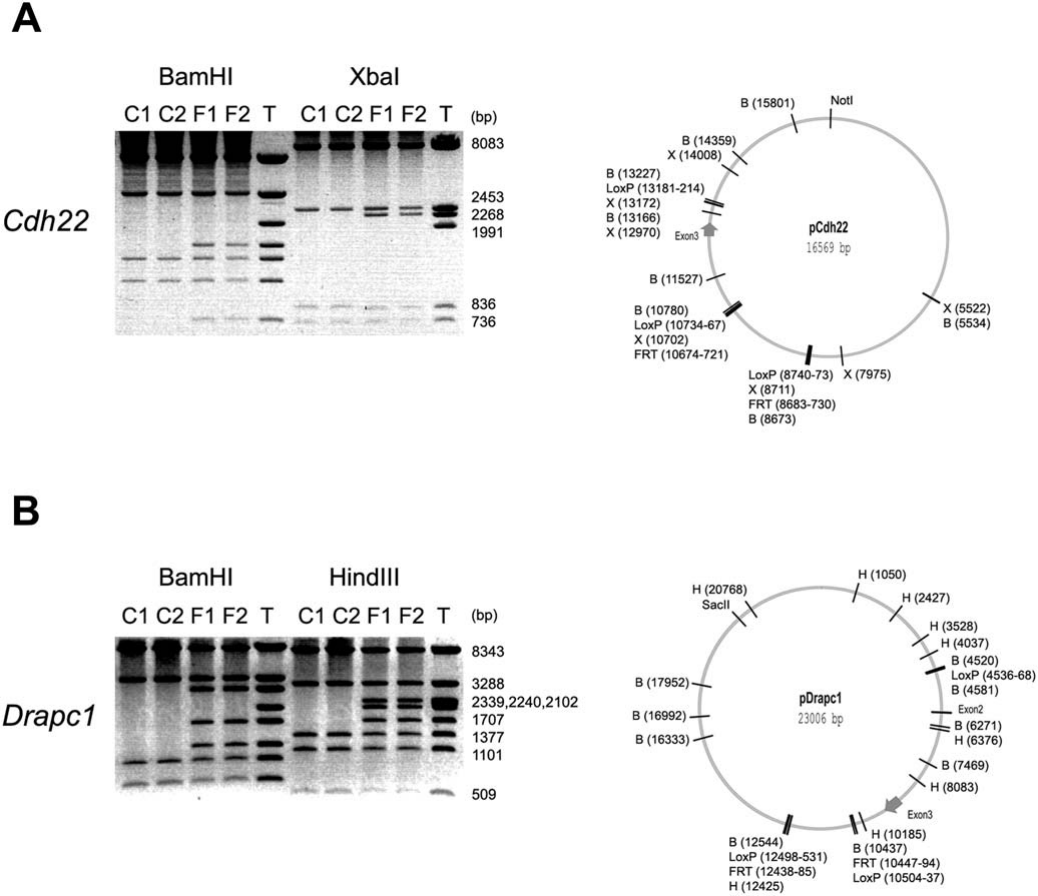
Verification of the functionality of the Cre/loxP and Flp/FRT site-specific recombination systems in the context of the gene targeting constructions. The final constructions were introduced into *E. coli* strains 294-Cre and 294-Flp, expressing Cre and FLP recombinase, respectively [28]. From each strain, two independent plasmid isolates, marked C1 and C2 (from the 294-Cre strain) or F1 and F2 (from the 294-Flp strain), were subjected to restriction analysis (on the left). On these analyses, the original targeting plasmid is marked with T. On the right are shown the respective plasmid maps with the relevant restriction enzyme as well as LoxP and FRT sites indicated (BamHI, XbaI and HindIII sites are indicated with B, X and H, respectively). (A) Analysis of the targeting construction for the *Cdh22* locus. As the size marker serves the XbaI digestion of the respective targeting construction. (B) Analysis of the targeting construction for the *Drapc1* locus. As the size marker serves the HindIII digestion of the respective targeting construction.

To verify experimentally the orientation and functionality of the loxP and FRT sites, the targeting constructs for *Cdh22* and *Drapc1* were introduced into *E. coli* strains 294-Cre and 294-Flp. These strains thermoinducibly express Cre and Flp recombinases, respectively, and can be used to monitor recombination-proficiency of plasmids [28]. In all four cases, site-specific recombination proceeded as expected (Figure 4), verifying authenticity of the constructions.

### Gene targeting in mouse ES cells

**Figure 5:**
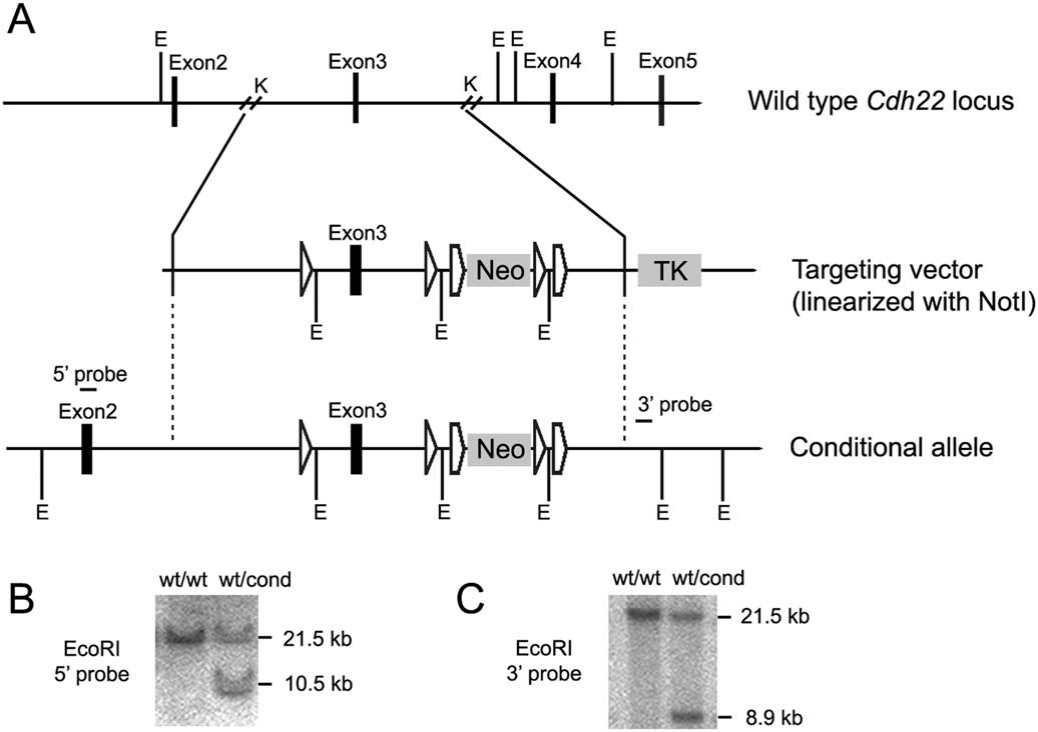
Targeting strategy for the conditional inactivation of the *Cdh22* gene. (A) Restriction maps of the mouse wild type *Cdh22* locus, the targeting vector, and the targeted allele. Relevant restriction sites: E, EcoRI; K, KpnI. Vertical dotted lines highlight homologous regions. (B) Southern analysis of genomic DNA from ES cells (EcoRI digestion), with the 5′-probe illustrating the 21.5 kb wild type (wt) and 10.5 kb targeted (cond) alleles. (C) Southern analysis of genomic DNA from ES cells (EcoRI digestion), with the 3′ probe illustrating the 21.5 kb wild type (wt) and 8.9 kb targeted (cond) alleles.

**Figure 6:**
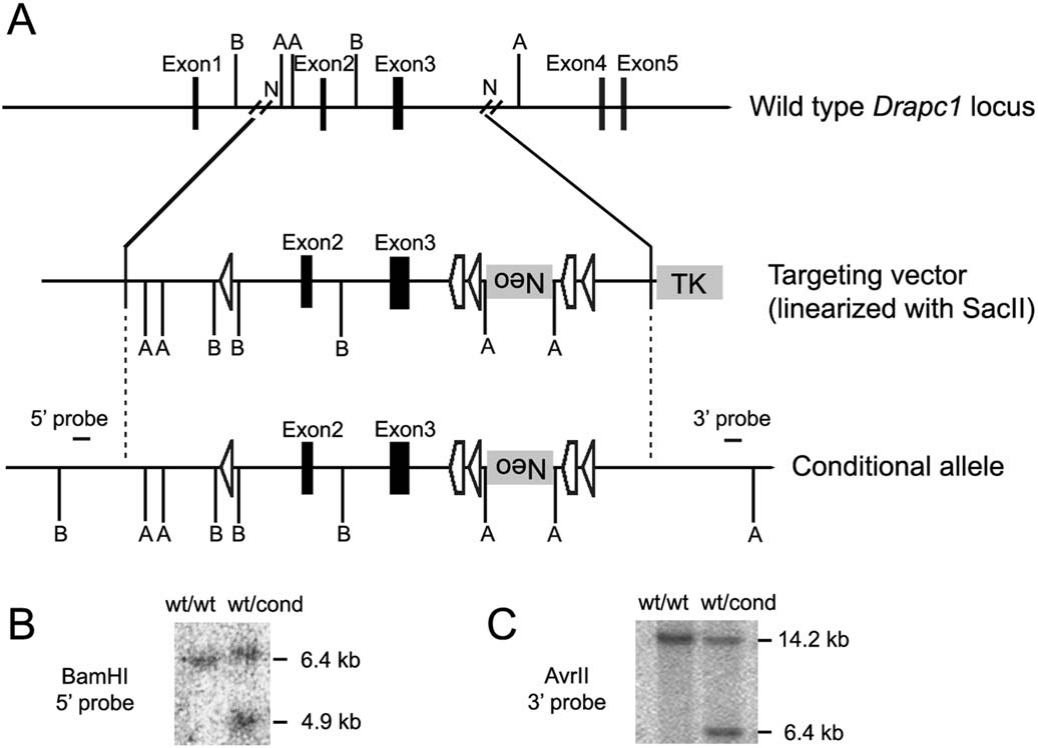
Targeting strategy for the conditional inactivation of the *Drapc1* gene. (A) Restriction map of the mouse *Drapc1* locus, the targeting vector and the targeted allele. Relevant restriction sites: A, AvrII; B, BamHI; N, NsiI. Vertical dotted lines highlight homologous regions. (B) Southern analysis of genomic DNA from ES cells (BamHI digestion), with the 5′ probe illustrating the 6.4 kb wild-type (wt) and 4.9 kb targeted (cond) alleles. (C) Southern analysis of genomic DNA from ES cells (AvrII digestion), with the 3′ probe illustrating the 14.2 kb wild type (wt) and 6.4 kb targeted (cond) alleles.

**Figure 7:**
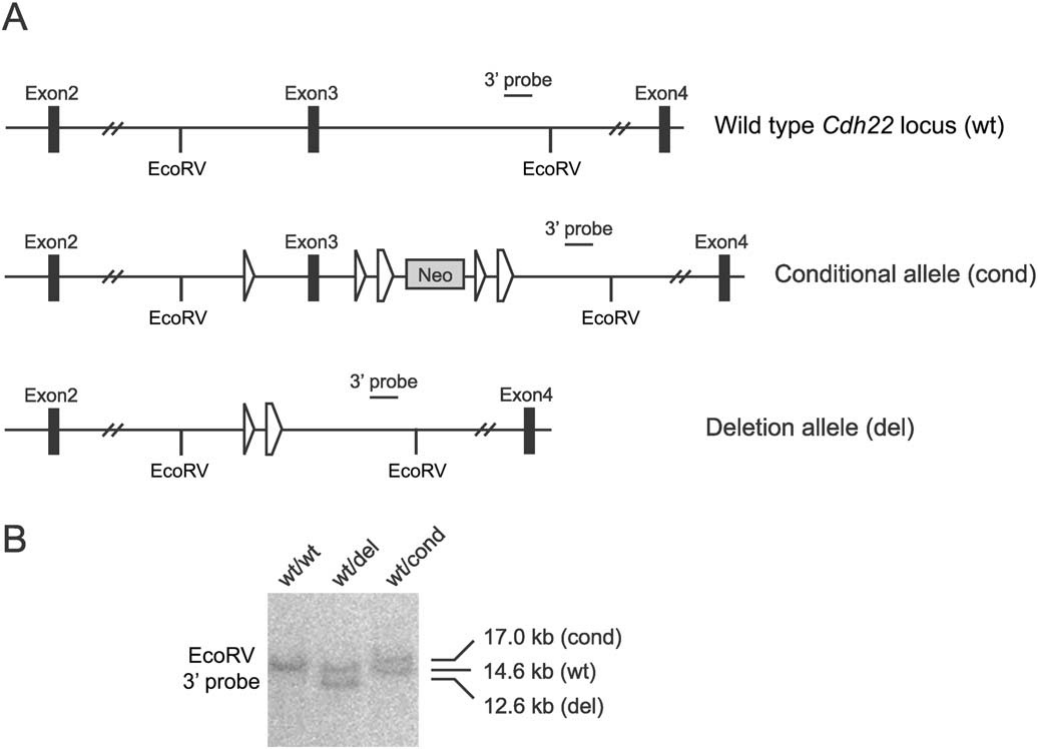
Comparison of wild type, conditional, and deletion alleles of the mouse *Cdh22* locus. (A) A schematic of wild-type (wt), conditional (cond), and Cre-recombined (del) *Cdh22* alleles. (B) Southern blot analysis of DNA isolated from the tails of mice carrying different *Cdh22* alleles (EcoRV digestion), with the 3′probe illustrating the 14.6 kb wild type (wt), 17.0 kb conditional (cond), and 12.6 kb deletion (del) alleles.

**Figure 8:**
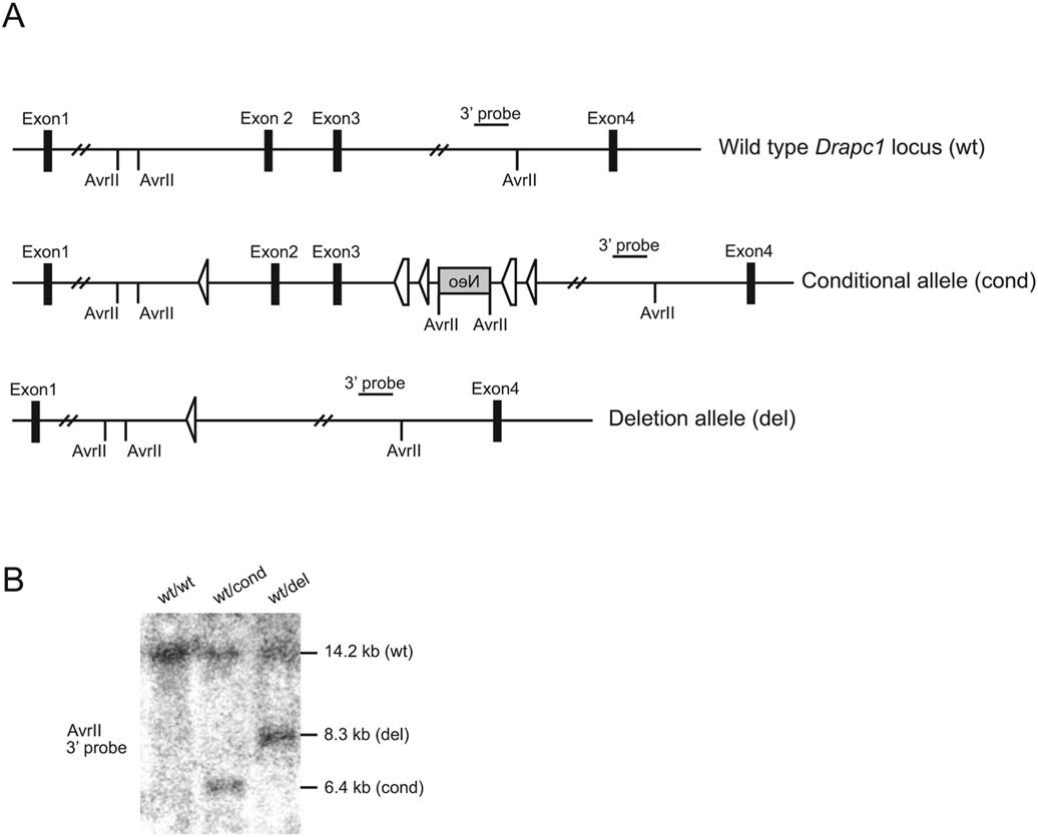
Comparison of wild type, conditional, and deletion alleles of the mouse *Drapc1* locus. (A) A schematic of wild-type (wt), conditional (cond), and Cre-recombined (del) *Drapc1* alleles. (B) Southern blot analysis of DNA isolated from the tails of mice carrying different *Drapc1* alleles (AvrII digestion), with the 3′probe illustrating the 14.2 kb wild type (wt), 6.4 kb conditional (cond), and 8.3 kb deletion (del) alleles.

The final constructions for *Cdh22* and *Drapc1* were linearized with NotI and SacII, respectively, and subsequently electrotransfected into mouse ES cells, selecting for the marker residing within the transposon (G418) and for the loss of the TK marker (ganciclovir). The selected ES clones were screened by Southern analysis with appropriate 5′ and 3′ probes to verify correct targeting. Six correctly targeted clones were identified for *Cdh22* gene and four for *Drapc1* gene, representing a targeting efficiency of 5.4 and 5.7%, respectively. Figures 5 and 6 show the restriction maps and Southern analyses of the wt as well as mutated *Cdh22^cond^* and *Drapc1^cond^* alleles. The correctly targeted ES cell clones were used to generate mouse chimeras, which transmitted the targeted *Cdh22^cond^* and *Drapc1^cond^* alleles through the germ-line (Figures 7 and 8). To demonstrate recombination between the LoxP sites and generate a null allele of *Cdh22*, mice carrying the *Cdh22^cond^* allele were crossed with mice carrying *PGK-Cre* transgene. In the double heterozygous (*Cdh22^cond^*/+; *PGK-Cre*/+) offspring, the *Cdh22^cond^* allele was efficiently converted by Cre-mediated recombination to *Cdh22^del^* allele, where the sequence between the LoxP sites was deleted (Figure 7). Similarly, also *Drapc1^del^* allele was produced (Figure 8).

## Discussion

We present a new strategy to produce gene targeting vectors for the generation of conditional knockout mutations in the mouse. To validate the methodology, we used the strategy to construct targeting vectors for *Cdh22* and *Drapc1* genes. These vectors were used for gene targeting in mouse ES cells, and the resulting conditional alleles were transmitted through the germ line.

The strategy includes a cloning vector with several desirable characteristics. For the selection against random integration in mouse ES cells, it contains the HSV TK gene. The array of multiple restriction endonuclease sites provides versatile possibilities for the choice of the genomic restriction fragments utilized and for the final linearization procedure prior to the delivery into ES cells. In addition, a homing endonuclease site is included for the linearization, and it can be used if none of the restriction enzyme sites is acceptable for that. The multicopy nature of the vector ensures a convenient plasmid production.

The strategy also includes two transposons, both of which contain the antibiotic-resistance cassette (Kan/Neo), selectable both in bacteria and mammalian cells and removable by Cre recombination. One of the transposons also contains a pair of FRT sites, enabling the excision of the resistance cassette by Flp-mediated recombination. Since the first report of the phage Cre/loxP system functioning in mammalian cells [29], both the Cre/loxP system and later the yeast Flp/FRT system have been shown to be effective in removing selectable markers in several types of mouse cells, including ES cells [30]–[33]. As the presence of a selectable marker within the modified locus can potentially influence the expression of the targeted gene [34], [35], a marker removal system is essential in any gene targeting strategy. In the described system, the most straightforward option to remove the positive selection cassette is the utilization of Flp recombination, as only two FRT sites are present in the modified locus. Although Cre recombination may also be used for the marker removal, the presence of three loxP sites necessitates a somewhat more meticulous screening phase. A marker removal in this system leaves behind a DNA segment that contains a pair of 50 bp Mu R-end sequences as an inverted repeat and an intervening sequence of 67 bp (Kan/Neo-loxP-Mu) or ∼120 bp (Kan/Neo-loxP-FRT-Mu) including the recombination signal(s). As these segments are relatively short, and they do not contain detectable splicing signals or polyadenylation sites, their presence in an intron is expected to have no or negligible effects on the level of gene expression from the modified loci. However, a degree of caution with this respect is warranted, as unpredictable locus-specific differences may possibly exist. We have shown that the marker-removed configurations are stable in plasmids, as no secondary rearrangements were observed in those plasmids that were isolated from recombinase-expressing *E. coli* strains (Figure 4). We have also shown that Cre-recombined alleles are stable in the mouse, as clear signals of stable loci were seen in the Southern analyses of the deletion allele mice (Figures 7 and 8). Given that Flp-recombined configurations are stable in plasmids (Figure 4), and because the respective alleles in mice would be very similar to those rearranged by Cre, we believe that Flp-recombined alleles are stable in mice, although this has not been verified experimentally.

In general, the use of DNA transposition strategies alleviates the requirement of finding appropriate restriction sites close to an exon of interest. In addition, with transposon strategies several constructions aimed at targeting different exons can be generated simultaneously. In Mu transposition, the target DNA sites are selected with a low stringency of sequence specificity [15], yielding essentially random distribution of integrations along longer regions of DNA [14], [16]. Accordingly, the target site selection of Mu transposition is optimally suited for the purpose established in this study.

In contrast to several published transposon protocols [6], [10], [11], the manipulation in our strategy is initiated directly from a BAC clone, and no prior subcloning of a particular restriction fragment is needed. During the transposon-assisted cloning step, a convenient double selection for the transposon (Kan^R^) and vector (Amp^R^) markers ensures that all cloned fragments surely contain the transposon. The probability of obtaining plasmid clones with two or more transposon insertions is very low because, in the reaction conditions used, most of the target DNA fragments do not experience even a single transposon integration [14], [16]. Nevertheless, because double integrations would be detrimental to the strategy, appropriate restriction analysis, or preferably sequencing with transposon-specific outwards-reading primers, should be used to ensure single-copy transposon integration. As illustrated in our study, sequencing also indicates the exact site of the transposon integration.

Following the initial transposon-assisted cloning step, the most convenient means to identify transposon-containing recombinant clones for further manipulation is arguably the use of PCR-based screening with sample pooling [36], [37]. Even in cases where the exon of interest resides in a sizeable restriction fragment, a suitable clone can be identified with a reasonable effort. In this study the targeted exon of the *Cdh22* gene was in a 9 kb KpnI fragment, a medium size fragment among those produced by the respective BAC clone. In this case, the screening of 300 colonies identified three suitable clones for further manipulation. In the *Drapc1* BAC clone, the two exons of interest were resident in a 15.1 kb NsiI fragment, which is one of the largest NsiI fragments of this BAC, generating a more challenging test case for the utility of the strategy. Even with this large fragment, two potentially suitable integrant clones were identified among 6,700 colonies with relative ease by the use of 40 pooled samples in the initial screening phase. Thus, it is straightforward to obtain desired transposon-containing recombinant plasmids with genomic inserts of 10–15 kb, a size range typically used for standard gene targeting projects. As a last step during the screening phase, we successfully used colony PCR, and this method proved to be highly effective and fast in identifying the final targeting constructions. The size range of PCR products that would identify a suitably located transposon is critically dependent on the resolution of the gel used and the performance of the DNA polymerase applied. In our study, PCR products ranging in size from 285 bp to 1,934 bp identified suitably located transposons (Figure 3).

Our strategy complements other targeting vector construction methods and offers certain key advantages: **(1)** The cloning vector and two transposons are generally usable and can be applied to modify any mouse gene. **(2)** No prior subcloning of BAC clone fragments or PCR amplification of marker cassettes flanked by homology regions is needed. **(3)** The first transposon can be integrated in one reaction into different locations over the entire length of the BAC clone, yielding a variety of possibilities for the choice of the targeted exon. **(4)** Both positive and negative selection markers are included. **(5)** Only one protein, MuA transposase, is needed. **(6)** Both loxP and FRT sites are present in the final gene targeting construct, allowing versatile possibilities for the gene modification in a variety of tissues and/or during different developmental stages.

Recently, two groups have applied transposon techniques for gene-targeting vector construction. Zhang *et al.* [10] used mini-Mu transposons, and Aoyama *et al.* [11] used a combination of two transposons, Tn*5* and Tn*7*. Many features in these two strategies do share similarities with our strategy. However, important differences also exist. For example, in the abovementioned two strategies, the modified gene region is initially subcloned into a plasmid vector. Negative selection was not employed in the Mu methodology, and two different transposon systems were used in the Tn*5*/Tn*7* methodology.

One of the advantages in our strategy, avoiding laborious initial cloning steps in gene-targeting vector construction, has been achieved also with recombineering, i.e. the use of homologous recombination in *E. coli* [5]. This method has been used to introduce loxP and FRT sites, and selection markers into genomic DNA [6]. Compared to transposon strategies, because of the PCR amplification step to insert homology regions, recombineering requires relatively long specific primers for each individual targeting construct. However, the advantage is that each particular locus can be modified very accurately as desired.

We have developed a fast and efficient Mu *in vitro* transposition-based procedure to construct targeting vectors directly from BAC clones without the need for prior subcloning. A general-purpose cloning vector and two custom-designed transposons provide a tool set for any conditional knockout project in mouse. The data indicate that the strategy described here is an easy, efficient, and versatile method for generating conditional knockout alleles. Furthermore, the procedure should be readily suitable for high-throughput targeting vector production.

## Materials and Methods

### Ethics statement

All the experiments involving animals were approved by the committee of experimental animal research of the University of Helsinki.

#### DNA techniques

Standard enzymes for DNA work [38] were from New England Biolabs. DyNAzyme II DNA polymerase (for cloning), Phusion DNA polymerase (for colony PCR), and MuA transposase were from Finnzymes. Enzymes were used as recommended by the suppliers. The BACs used were screened from the 129S6/SvEvTac mouse BAC library RPCI-22 (BACPAC Resources). Qiagen kits were used for DNA isolation. Standard DNA techniques were performed as described [38]. The colony PCR method was modified from a previously published protocol [39]. Briefly, a single colony was picked from a selection plate and suspended in 50 µl of water. One microliter of this suspension was then used as a template in PCR amplification, using Phusion DNA polymerase according to the recommendations of the supplier. Each PCR amplification included one transposon-specific and one locus-specific primer (Figure 3).

#### Cloning vector

The herpes simplex virus (HSV) thymidine kinase (TK) gene cassette including the mouse phosphoglycerate kinase (PGK) promoter and terminator was cloned from plasmid pPNTloxP [40], as an EcoRI-HindIII fragment, into plasmid pUC19 (New England Biolabs) that had been cleaved with the same two enzymes, yielding pHTH21. A polylinker was then generated by annealing and ligating oligonucleotides HSP478 through HSP484 (Table 1). The polylinker was PCR-amplified using primers HSP485 and HSP486; and cloned, as an EcoRI fragment, into the EcoRI site of pHTH21. Finally, one of the EcoRI sites was eliminated by partial digestion, end-filling with Klenow enzyme, and ligation to generate plasmid pHTH22 (Figure 1A and 1B).

**Table 1:** Oligonucleotides used in this study.

Oligonucleotide	Sequence	Usage
HSP478	5′-CATCTATGTCGGGTGCGGAGAAAGAG	Linker construction, Watson strand
HSP479	5′-GTAATGAAATGGCAGCGATCGCGCGGCCG	Linker construction, Watson strand
HSP480	5′-CCACCGGTGGGCGCGCCGGTACCGTTAACATCGATATG	Linker construction, Watson strand
HSP481	5′-CATGGATCCGTCGACTCTAGAG	Linker construction, Watson strand
HSP482	5′-CTCTAGAGTCGACGGATCCATGCATATCGATGTTAAC	Linker construction, Crick strand
HSP483	5′-GGTACCGGCGCGCCCACCGGTGGCGGCCGCGCGATCG	Linker construction, Crick strand
HSP484	5′-CTGCCATTTCATTACCTCTTTCTCCGCACCCGACATAGATG	Linker construction, Crick strand
HSP485	5′-CCGGCCGAATTCATCTATGTCGGGTGCGG	Linker amplification, forward
HSP486	5′-CCGGCCGAATTCTCTAGAGTCGACGGTACC	Linker amplification, reverse
HSP474	5′-CTAGGAAGTTCCTATTCCGAAGTTCCTATTCTCTAGAAAGTATAGGAACTTC	1. FRT site construction
HSP475	5′-CTAGGAAGTTCCTATACTTTCTAGAGAATAGGAACTTCGGAATAGGAACTTC	1. FRT site construction
HSP476	5′-AAGCTTTTAATTAAGAAGTTCCTATTCCGAAGTTCCTATTCTCTAGAAAGTATAGGAACTTCAT	2. FRT site construction
HSP477	5′-GAAGTTCCTATACTTTCTAGAGAATAGGAACTTCGGAATAGGAACTTCTTAATTAAAAGCTTAT	2. FRT site construction
HSP422	5′-CCTCTTCGCTATTACGCCAG	Vector-specific sequencing
HSP506	5′-AAGCGCATGCTCCAGACTGC	Vector-specific sequencing
HSP430	5′-ACATTGGGTGGAAACATTCC	Tn-specific PCR
HSP432	5′-CCCCGGGCGAGTCTAGGGCCGC	Tn-specific sequencing
N2	5′-GCGTTTTTCGTGCGCCGC	Tn-specific PCR
1B5′	5′-CCGGGCGAGTCTAGGGCCGC	Tn-specific PCR
1B3′	5′-GGGAACACAGAGAGACCCAGAAGC	*Cdh22* -specific PCR
2B5′	5′-ATCAACGACAGTGAACCACG	*Cdh22* -specific PCR
loxP1seq5′	5′-CACTGGTCGGCCTTCTTCAGG	*Cdh22*-specific sequencing
HSP507	5′-CGAGAATCAGAACCTAGCTTGC	*Drapc1*-specific PCR
HSP515	5′-GGACTATGGAGACCCTTGC	*Drapc1*-specific sequencing
HSP517	5′-ACAGTGGGAAGGAACAATGC	*Drapc1-*specific sequencing
HSP518	5′-GAGCATACAAAAGCGGATGG	*Drapc1-*specific PCR
HSP524	5′-GGCAGAGCCTTGCAGGGAG	*Drapc1*-specific sequencing

#### Transposons

Two related mini-Mu transposons (Figure 1C) were constructed using standard PCR and cloning procedures. Similar to the transposons in earlier studies [14], [20], these transposons were produced as a segment of their pUC19-derived carrier plasmids. Plasmids pHTH19 and pHTH24 carry Kan/Neo-loxP-Mu and Kan/Neo-loxP-FRT-Mu transposons, respectively. Both of these transposons contain the selection cassette from plasmid pIRES2-EGFP (Clontech), including two promoters, prokaryotic (p_bact_) and eukaryotic (p_SV40_), in addition to the Kan/Neo resistance-encoding gene from Tn*5* and polyadenylation signals from the HSV TK gene. The Kan/Neo marker gene can be used both with *E. coli* (kanamycin selection) and mammalian cells (G418 selection). Flanking the selection cassette, the transposons contain a transposon-specific set of Cre and Flp recombination sites, and the extreme transposon termini each include 50 bp from Mu R-end as an inverted repeat to provide critical MuA transposase binding sites. The transposons were released from their carrier plasmids by BglII digestion and purified using anion exchange chromatography as described [14].

#### Introduction of Kan/Neo-loxP-Mu

The initial *in vitro* transposition reaction mixture (20 µl) contained 0.185 pmol (250 ng) transposon DNA (Kan/Neo-loxP-Mu), 500 ng target BAC DNA digested with an appropriate restriction enzyme, 2.46 pmol (176 ng) MuA, 25 mM Tris-HCl, pH 8.0, 0.05% (w/v) Triton X-100, 10% glycerol (v/v), 120 mM NaCl and 10 mM MgCl_2_. Reaction was carried out for 4 h at 30°C. The resulting transposon-containing fragment pool, purified by phenol and chloroform extractions and ethanol precipitation, was ligated to 4 µg of the plasmid pHTH22 linearized with the same enzyme as was used for the initial BAC digestion. The ligation mixture was purified by phenol and chloroform extractions and ethanol precipitation, and dissolved in 20 µl of water. Several aliquots (4 µl) were electroporated into electrocompetent DH10B cells (50 µl) prepared as described [14] using Genepulser II (Bio-Rad) and 0.2 cm electrode spacing cuvettes (Bio-Rad) as described [14]. Insert-containing plasmid clones were selected on LB/ampicillin/kanamycin plates and screened by colony PCR for the presence of the transposon in the desired DNA region using a pair of appropriate primers, one transposon-specific and one locus-specific. The exact location of each identified transposon was confirmed by initial restriction analysis and subsequent sequencing.

#### Removal of Kan/Neo selection marker

The selected plasmid was electroporated into strain 294-Cre [28] for *in vivo* recombination, after which the plasmid was reisolated; recombination between the two loxP sites was confirmed by restriction analysis and sequencing. To prevent any potential Cre-mediated further rearrangements and for the production of good quality plasmid DNA for further manipulation, the accurately Cre-recombined plasmid was introduced into *E. coli* DH10B for isolation. Note! PCR-based DNA sequencing strategies may not yield sequencing reads across two Mu ends in inverse relative orientation, possibly due to intramolecular hybridization problems. Good quality reads can be obtained by linearizing the DNA between Mu ends prior to sequencing reactions (e.g. using EcoRI or XbaI).

#### Introduction of Kan/Neo-loxP-FRT-Mu

Mu transposition complexes were initially assembled at 30°C in a 20 µl reaction volume for 2 h. The assembly mixture contained 4.7 µl (1.1 pmol; 1590 ng) transposon DNA (Kan/Neo-loxP-FRT-Mu), 4 µl 5× assembly buffer (750 mM Tris-HCl, pH 6.0, 0.125% (w/v) Triton X-100, 750 mM NaCl and 0.5 mM EDTA), 50% (v/v) glycerol, and 1 µl (4.9 pmol; 350 ng) MuA. The resulting complexes (1 µl) were added to the mixture (18 µl), containing 0.55 pmol plasmid DNA as a target, 25 mM Tris-HCl, pH 8.0, 110 mM NaCl, 0.05% Triton X-100 (w/v), 10% glycerol, and incubated for 5 min at 30°C to allow target capture. MgCl_2_ (1 µl) was then added to the final concentration of 10 mM and incubation was continued for 2 min in a total volume of 20 µl. The transposition reaction mixture was electroporated into *E. coli* DH10B electrocompetent cells, and transposon-containing clones were selected on LB/ampicillin/kanamycin plates and screened by colony PCR. For each candidate clone, the location and orientation of the transposon were determined by sequencing.

#### Gene targeting

Targeting constructions were linearized and electroporated into mouse embryonic stem cells [27]. Desired clones were cultured using a medium containing G418 (136 µg/ml) and ganciclovir (2.64 µM) for positive and negative selection, respectively. The selected ES clones were screened by Southern blotting with appropriate 5′ and 3′ probes to verify correct targeting. Recombinant ES cell clones were used for morula aggregations to produce chimeras, which transmitted the mutant alleles through the germ line.

#### Cre-recombination in mice

The mice carrying the targeted alleles were crossed with PGK-Cre mice [41]. The offspring was screened by Southern blotting with appropriate 3′ probes using genomic DNA isolated from tails.
